# Identification of Genetic Variants *via* Bacterial Respiration Gas Analysis

**DOI:** 10.3389/fmicb.2020.581571

**Published:** 2020-11-16

**Authors:** Naoki Koga, Takuro Hosomi, Martijn Zwama, Chaiyanut Jirayupat, Takeshi Yanagida, Kunihiko Nishino, Seiji Yamasaki

**Affiliations:** ^1^School of Pharmaceutical Sciences, Osaka University, Osaka, Japan; ^2^Graduate School of Engineering, The University of Tokyo, Tokyo, Japan; ^3^Institute of Scientific and Industrial Research, Osaka University, Osaka, Japan; ^4^Institute for Materials Chemistry and Engineering, Kyushu University, Fukuoka, Japan; ^5^Institute for Advanced Co-Creation Studies, Osaka University, Osaka, Japan

**Keywords:** bacteria, gas, indole, gas chromatography-mass spectrometry, *tnaAB*, *Escherichia coli*

## Abstract

Indole is a signal molecule derived from the conversion of tryptophan, and it is present in bacterial respiratory gas. Besides influencing bacterial growth, indole exhibits effects on human health, including a positive effect on inflammation and protection against pathogens. However, a high fecal indole concentration (FIC) can suggest an unbalanced gut flora or the presence of certain pathogens. To analyze the indole produced by bacteria, its collection and detection is required. Traditional methods usually require centrifugation of liquid bacterial culture medium and subsequent extraction of indole from the medium or partial purification of indole from fecal samples (e.g., by distillation or extraction). In this study, we demonstrate the possibility of identifying gas contents directly from bacteria, and we distinguish the difference in species and their genetics without the need to centrifuge or extract. Using an absorbent sheet placed above a liquid culture, we were able to collect gas content directly from bacteria. Gas chromatography-mass spectrometry (GC-MS) was used for the analysis. The GC-MS results showed a clear peak attributed to indole for wild-type *Escherichia coli* cells (MG1655 and MC4100 strains), whereas the indole peak was absent in the chromatograms of cells where proteins, part of the indole production pathway from tryptophan (TnaA and TnaB), were not expressed (by using *tnaAB*-deleted cells). The indole observed was measured to be present in a low nmol-range. This method can distinguish whether the bacterial genome contains the *tnaAB* gene or not and can be used to collect gas compounds from bacterial cultures quickly and easily. This method is useful for other goals and future research, such as for measurements in restrooms, for food-handling facilities, and for various applications in medical settings.

## Introduction

Human gastrointestinal tract bacteria can survive in oxygen-deprived (anaerobic) conditions. These microbes produce signal substances, which not only regulate bacterial growth, but also influence human health by affecting biological functions (Berstad et al., 2015). Indole is the main metabolite produced by enteric bacteria from tryptophan, a quorum-sensing compound (Kim and Park, 2013) and exhibits a major influence on host metabolism (Chimerel et al., 2014). We previously reported the contribution of multidrug efflux pumps expressed in bacteria to the removal of indole-derivative compounds under anaerobic conditions (Hirakawa et al., 2004; Zhang et al., 2011). Indole compounds are released into the natural environment of anaerobic bacteria in the human gut, as a component of their respiratory gas. Indole regulates various bacterial functions, including drug resistance, virulence, and biofilm formation (Lee et al., 2009; Lee and Lee, 2010; Nikaido et al., 2012). As indole is believed to exhibit a significant influence on host metabolism, it directly impacts human health. Sonowal et al. (2017) studied the effects of indole in geriatric worms, flies, and mice, in which indole extended health spans, suggesting that indole helps to retain a “young gene expression profile” in animals. Additionally, indole produced by intestinal bacteria was found to relieve inflammation of the liver in mice (Beaumont, 2018).

Some of the health effects of indole and indole derivatives were linked to the activation of the aryl hydrocarbon receptor by acting as agonists (Hubbard et al., 2015; Puccetti et al., 2018), an action which is also directly linked to protection against pathogenic bacteria (Romani et al., 2014). The effect of microorganisms on human health has been of increased interest to researchers (Cani, 2019). However, as well as its beneficial properties, indole also was suggested to be potentially harmful to the human body. A high concentration of the indole derivative, indoxyl sulfate, was related to chronic kidney disease and vascular disease in humans (Zhang and Davies, 2016).

As indole is therefore involved in human health, developments in its collection and detection methods are important. Examples of procedures used to measure indole produced by bacteria include steam distillation (Happold and Hoyle, 1934) and gas chromatography-mass spectrometry (GC-MS; Jensen and Jensen, 1994). These methods usually require the following steps: centrifugation of the liquid culture medium with cultured bacteria, and extraction of indole from the medium using an organic solvent, such as hexane or chloroform (more details regarding different techniques are mentioned in the Discussion section). These steps are very time-consuming, and, in particular, the extraction step often requires high volumes of reagent and is easily influenced by the experimenter. In addition, the identification of bacteria is usually based on biochemical reactions, such as in the analytical profile index (API), or genetic screening, which checks RNA‐ or DNA-specific sequences. Currently, no practical methods that obtain bacterial profiles directly from samples and measure them easily and quickly exist. Therefore, in the present study, we aimed to develop a single-step method to identify genetic features based on bacterial respiratory gas. We demonstrate that indole gas, gathered directly from samples, can be collected easily, without centrifuging the samples or extracting indole from the medium. We were able to clearly distinguish the genomic traits of the different bacterial strains used. If we can distinguish the species of bacteria and their genetics based on the compounds that the bacteria produce, the method can be applied to various settings, including the medical field and food hygiene management.

## Materials and Methods

### Strains and Conditions

The *Escherichia coli* strains used in this study were MG1655 and MC4100 (reference NKE104 and NKE259, respectively; Table 1). They were prepared from stocks of MG1655 (Blattner et al., 1997) or MC4100 (Casadaban, 1976). The gene-deletion strains used in this study are NKE256 (MG1655∆*tnaAB*) and NKE258 (MC4100∆*tnaAB*; Hirakawa et al., 2004). MG1655∆*tnaAB* was created by the method described by Link et al. (1997) with primers *tnaAB*-No, *tnaAB*-Ni, *tnaAB*-Ci, and *tnaAB*-Co (Table 2). With 10% skim milk for long-term storage, the bacterial strains were stored at −80°C. The strains were cultured in Luria-Bertani (LB) broth (BD Difco) at 37°C overnight. The primers used to create the *tnaAB*-deletion strains and their sequences are provided in Table 2.

**Table 1:** Strains and plasmids used in this study.

Strains	Description	Source or reference
***Escherichia coli* strains**		
NKE104	MG1655	Blattner et al., 1997
NKE259	MC4100	Casadaban, 1976
NKE256	MG1655*ΔtnaAB*	Hirakawa, unpublished
NKE258	MC4100Δ*tnaAB*	Hirakawa et al., 2004

Wild-type *E. coli* strains MG1655 and MC4100 were used to create the tnaAB knock-out mutants (explained in Methods and Table 2).

**Table 2:** Primers and sequences used in this study.

Primers	Sequence (5'–3')
*tnaAB*-No	cgcggatcctttctccagcttctgtattgg
*tnaAB*-Ni	cacgcaataaccttcacactccaaatttataaccatttattttaattacagtgatccctg
*tnaAB*-Ci	gttataaatttggagtgtgaaggttattgcgtgtaaatccttcaagaagccagccattcg
*tnaAB*-Co	cgcgtcgacgacagcactttagcccgacg

Primers used for the gene-deletion of tnaAB in MG1655 and MC4100 *E. coli* cells. Primer names in accordance with the method description by Link et al. (1997).

### Collection of Gas Ingredients From Live Microbes

**Figure 1:**
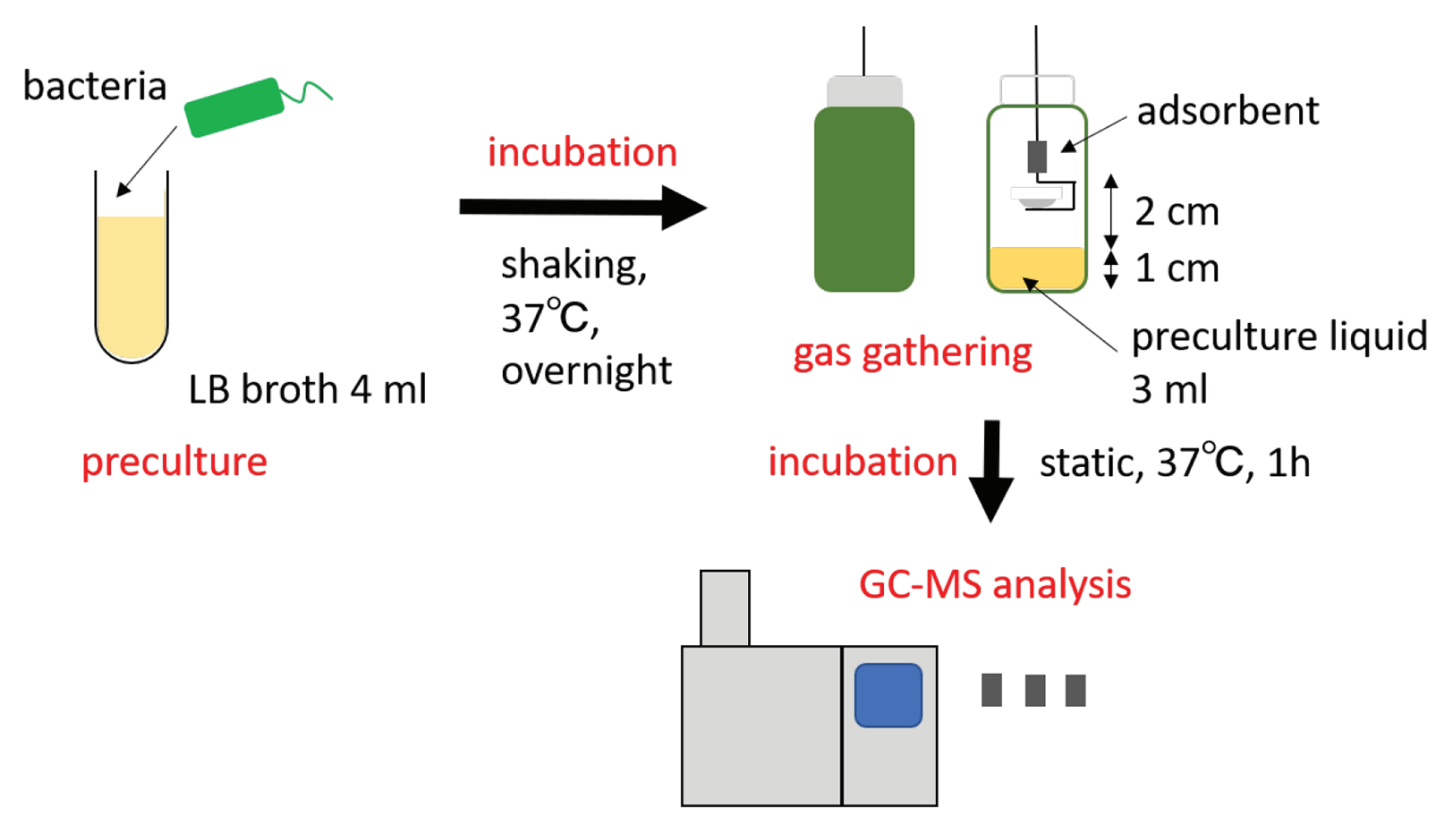
Overview of the indole analysis method applied in this study. Bacteria cultured overnight were transferred to a vial, where the bacterial gas was absorbed above the culture. The absorbent was then analyzed by gas chromatography-mass spectrometry (GC-MS).

The procedure overview can be seen in Figure 1. Each strain was cultured in 4 ml LB broth at 37°C overnight. The following day, stainless steel cups (Eco-Cup SF, Frontier Laboratories Ltd., Japan) were attached to a wire (Eco-Stick DF, Frontier Laboratories Ltd., Japan) equipped with a thermal desorption adsorbent (MonoTrap RGPS TD, GL Sciences B.V., The Netherlands). The wire was pierced through the covers of the bottles (septa, blue PTFE/white silicone, Supelco). The amber glass vials (screw-top vial, Supelco) contained 1 cm of bacterial liquid culture (3 ml volume). The adsorbent was placed 2 cm above the culture, and it was immediately cultured at 37°C for 1 h, without shaking. The vapor pressure of indole is high even at room temperature (0.016 hPa at 25°C); therefore, the effect of temperature disturbance is considered to be quite small. After collecting the gas ingredients, the adsorbents were put into 1.5 ml anti-static tubes and stored at 4°C until further use. As a negative control, the same protocol was performed in LB media without bacteria. Each experiment was repeated at least three times.

### GC-MS Analysis of Bacterial Respiratory Gas

Before GC-MS analyses, the gas-adsorbed adsorbents prepared in the section of “Collection of gas ingredients from live microbes” were transferred to a liner (diameter: 4.5 mm and length: 80 mm) with tweezers. The liner was immediately set to an inlet port of a gas chromatography-mass spectrometer (GCMS-QP2020A, Shimazu Corporation, Japan). The inlet port temperature was controlled by an inlet-temperature control system (OPTIC-4 inlet, GL Sciences B.V., The Netherlands). First, the inlet temperature was kept at 35°C. For the gas-desorption from the adsorbent, the injection port was heated to 250°C with a rate of 5°C/s, and kept at this temperature until the end of the measurements. The desorbed gas was flowed into a capillary column (SLB-IL60 capillary GC column, Supelco) and detected by the MS detector (Figure 1). The column oven temperature was first kept at 40°C for 6 min, followed by heating to 280°C with a rate of 5°C/min, and then kept at the temperature for 5 min. The amount of desorbed indole was estimated by comparing the peak area of indole with that of a calibration standard.

## Results

To detect and check for indole in gaseous state, we used wild-type *E. coli* strains and strains lacking the *tnaAB* genes (see Materials and Methods), which encode tryptophan-specific transporter TnaB and tryptophanase TnaA (which converts tryptophan into indole and pyruvate). In addition, considering the difference in pedigrees, we performed the experiment for two *E. coli* K12 strains, MC4100, and MG1655.

**Figure 2:**
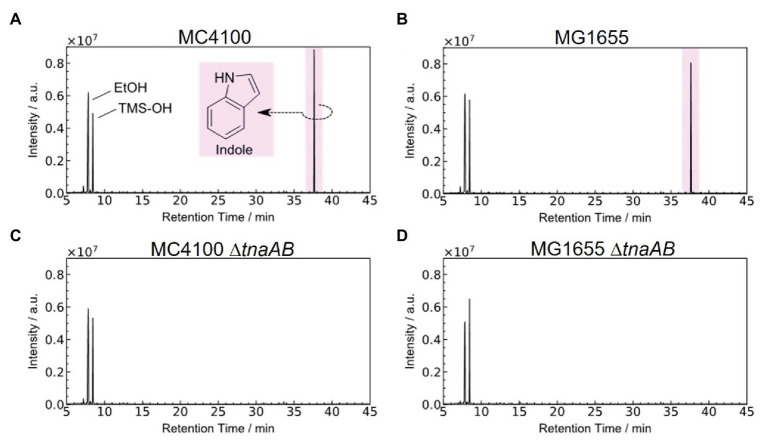
Typical GC-MS chromatograms of the gas released from different *Escherichia coli* strains. Chromatograms of the gas present in the absorbent for all four strains, **(A)** MC4100, **(B)** MG1655, **(C)** MC4100∆*tnaAB*, and **(D)** MG1655∆*tnaAB*. The detected organic species were identified from their mass/charge ratio. Trimethylsilanol is considered to be derived from the adsorbent material. Each experiment was repeated at least three times. EtOH, ethanol; TMS-OH, trimethylsilanol. Data shown is one of the results. Repeats gave similar results (Supplementary Figure S1). Indole was obtained and measured in a low nmol-range (Supplementary Table S1).

**Figure 3:**
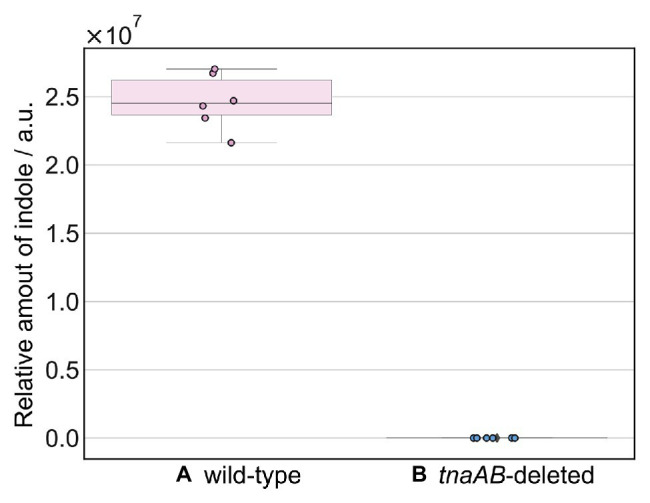
Box plot of the relative amounts of indole. The amount of indole produced by **(A)** wild-type strains (MC4100 and MG1655) and **(B)** *tnaAB*-deleted strains (MC1655∆*tnaAB* and MG1655∆*tnaAB*). The amount of indole was calculated from the peak area at 37.6 min retention time in the GC-MS spectrum. The error bars represent the 95% confidence boundaries of the measured values. GC-MS analysis of each strain was repeated at least three times.

Figure 2 shows the GC-MS chromatogram results for the biogas collected from each of the four strains: MC4100, MG1655, and their *tnaAB*-deletion counterparts. Two sharp peaks at early retention times were observed in the all chromatograms, and they were attributed to ethanol (EtOH; from the vaporized EtOH used for disinfection) and trimethylsilanol (TMS-OH; from the adsorbent). For both wild-type strains, a clear, sharp peak appeared at 37.6 min retention time (Figures 2A,B). Based on the mass fragment pattern using mass spectrometry, this peak was attributed to indole. In the chromatograms of both MC4100 and MG1655, the indole peak showed a significantly higher intensity compared to any other bacterial volatile compound. The indole peak was completely absent in the chromatograms for the *tnaAB*-deletion strains (Figures 2C,D). This sharp contrast can be directly explained by the lack of indole transporter and indole production enzymes in the *tnaAB*-deleted strains. Repeated experiments proved that this difference was statically significant (Supplementary Figure S1). A box plot of the different measurements is shown in Figure 3. The amount of indole could also determine quantitatively by using a standard curve, and was 1.5 and 1.4 nmol for MC4100 and MG1655 respectively, while 0 nmol for the knock-out cells (Supplementary Table S1).

## Discussion

During the past century, various methods were employed to measure the indole produced by bacteria (Bergman, 1917). For example, Happold and Hoyle (1934) extracted indole from bacterial culture media using multiple distillation steps. Jensen and Jensen (1994) measured indole present in bacterial culture media by GC-MS. These methods are still frequently used today. However, in order to prepare the measurement samples, these methods require an additional step to extract indole from the bacterial media. Other methods were also developed. Darkoh et al. (2015) describe a method to quantify the amount of indole in biological samples using the hydroxylamine-based indole assay, which can detect indole specifically (but not indole analogs). While this assay is rapid and precise in quantitation, it lacks the ability to assess indole from bacterial gas and also requires extraction from the samples.

In this study, we gathered indole gas directly from live microbes, from the atmosphere above a liquid bacterial medium and then analyzed it using GC-MS. Figure 2 shows that the wild-type K12 *E. coli* strains (MC4100 and MG1655) produced a sharp, clear indole peak, while the *tnaAB*-deleted strains did not produce this peak. The results illustrated in Figure 3 show that the indole produced by the wild-type strains and caught by the absorbent was in ample abundance for detection, and it produced a significantly higher total amount compared to the *tnaAB*-deleted strains. The expectation exists that a *tnaAB*-deletion strain does not produce indole (Kobayashi et al., 2006), and these strains were therefore used as a negative control to verify the indole peak present in the wild-type strains’ chromatograms. Based on these results, we demonstrated that, with this quick and easy method, detecting indole and obtaining a clear and sharp peak in the chromatograms of samples from indole-producing bacteria is possible. The MonoTrap system is in essence miniaturized solid-phase microextraction (SPME), which we chose for its relatively fast analytical properties and compared to other SPME methods, and does not require solvents which may pollute the samples, and can if needed be used with both chemical and thermal desorption. In addition, the MonoTrap allowed for a small sample size to be analyzed, due to its high surface area (Dugheri et al., 2020).

As the collection of indole in this method is easy and fast, it can be used to analyze a large number of samples and obtain time-series data. The method could also potentially be automated. As mentioned earlier, indole is a metabolite produced by enteric bacteria. If indications of fecal indole concentrations (FICs) can be rapidly determined from gaseous states, they could be used as markers to detect pathogenic microorganisms or an unhealthy microflora. Zhu et al. (2010) measured the volatile organic components (VOCs) that different species of bacteria produced by second electrospray ionization mass spectrometry (SESI-MS), and they identified bacteria by looking for distinct patterns in VOC detection. Additionally, recently, researchers suggested that FICs can act as biomarkers to indicate bacterial infections (Chappell et al., 2016). A previous study (Darkoh et al., 2015) suggested a specific range of FICs that are valid for the majority of unhealthy individuals. Indole detection and quantification devices could be used as sensors in restrooms, in food-handling facilities, or in medical settings, where rapid identification of bacterial species is a major advantage. Future research is needed to verify the quantification of indole present in bacterial respiratory gas and its correlation to individuals’ health and gut flora. We believe our quick indole collection method could be used in the future development of detection devices and therefore contribute to the improvement of human health.

## Data Availability Statement

The original contributions presented in the study are included in the article/Supplementary Material, further inquiries can be directed to the corresponding authors.

## Author Contributions

NK, KN, and SY performed the molecular biological and biochemical experiments. TH, CJ, and TY performed GC-MS analysis. NK, KN, MZ, and SY wrote the manuscript. All authors contributed to the article and approved the submitted version.

### Conflict of Interest

The authors declare that the research was conducted in the absence of any commercial or financial relationships that could be construed as a potential conflict of interest.
