# Supplementary material for: Identification of Genetic Variants via Bacterial Respiration Gas Analysis
Source: Front Microbiol. 2020 Nov 16;11:581571. doi: 10.3389/fmicb.2020.581571 (PMC7701088; doi:10.3389/fmicb.2020.581571)
Supplement: Supplementary file 1 [file Data_Sheet_1.pdf]

Supplementary Material

Identification of Genetic Variants via Bacterial Respiration Gas Analysis

Naoki Koga, Takuro Hosomi, Martijn Zwama, Chaiyanut Jirayupat, Takeshi Yanagida, Kunihiko Nishino*, Seiji Yamasaki*

*** Correspondence:** Kunihiko Nishino. nishino@sanken.osaka-u.ac.jp
Seiji Yamasaki. seiji37@sanken.osaka-u.ac.jp

Supplementary Figures

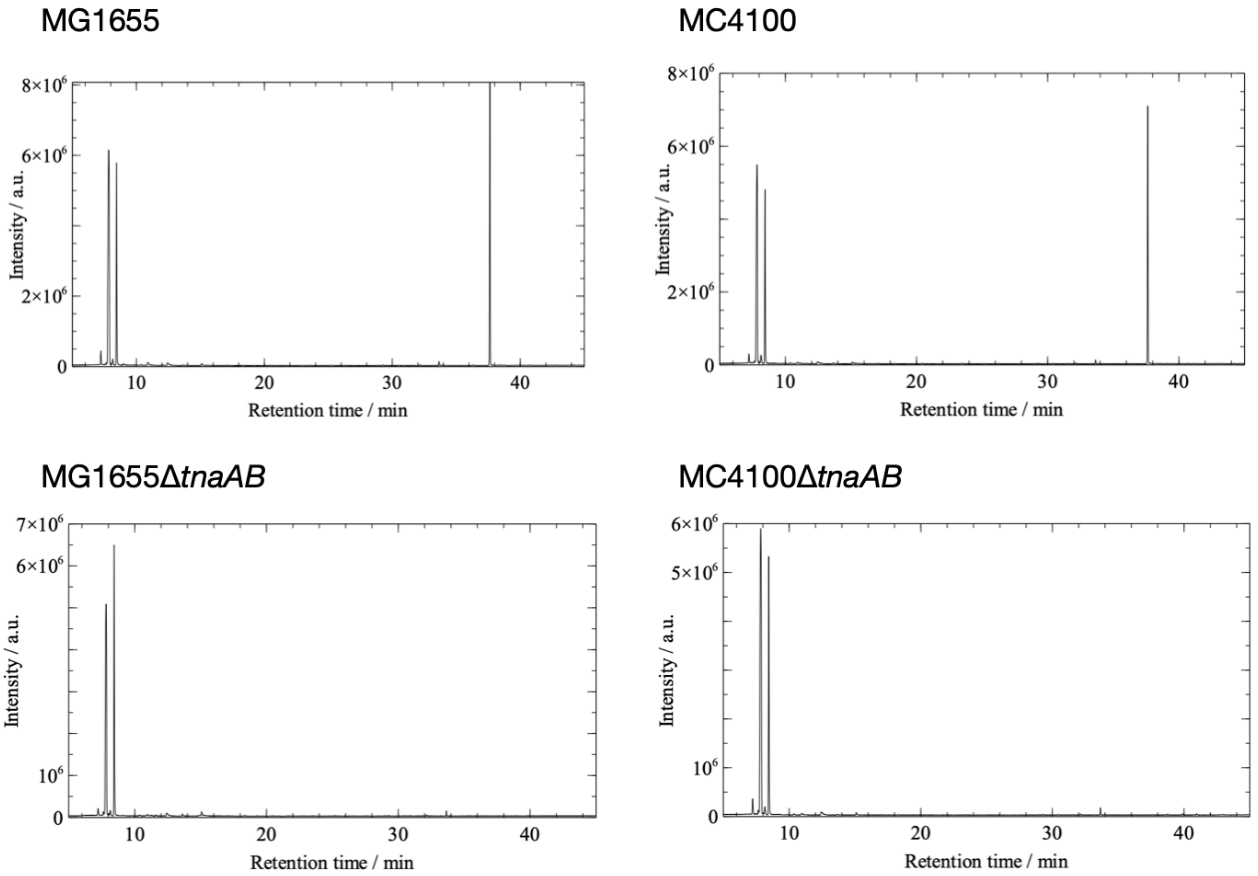

Supplementary Figure 1. Repeats of the GC-MS chromatograms of the gas released from different *Escherichia coli* strains. Chromatograms of the gas present in the absorbent for all four strains MC4100, MG1655, MC4100Δ*tnaAB*, and MG1655Δ*tnaAB*. The detected organic species were identified from their mass/charge ratio.

Supplementary Table 1

	MC4100	MG1655	MC4100 *ΔtnaAB*	MG1655 *ΔtnaAB*
Amount of adsorbed indole (nmol)	1.5	1.4	0	0

Adsorbed amount of indole calculated by GC-MS analyses using a calibration standard.
